# Frequency of Impulsive-Compulsive Behavior and Associated Psychological Factors in Parkinson’s Disease: Lack of Control or Too Much of It?

**DOI:** 10.3390/medicina59111942

**Published:** 2023-11-02

**Authors:** Alexandros Kapsomenakis, Dimitrios Kasselimis, Emily Vaniotis, Anastasia Bougea, Christos Koros, Athina Maria Simitsi, Leonidas Stefanis, Constantin Potagas

**Affiliations:** 1 1st Department of Neurology, Eginition Hospital, School of Medicine, National and Kapodistrian University of Athens, 10679 Athens, Greeceannita139@yahoo.gr (A.B.);; 2 Department of Psychology, Panteion University of Social and Political Sciences, 17671 Athens, Greece

**Keywords:** Impulsive-Compulsive Disorders, impulsive-compulsive behavior, Parkinson’s Disease, personality traits, activity, impulsivity

## Abstract

*Background and Objectives:* Impulse Control Disorders (ICDs) including pathological gambling, hypersexuality, compulsive eating, compulsive buying, and other related behaviors are well-known distinct non-motor symptoms in Parkinson’s Disease (PD). Some large-scale studies present a prevalence of at least 10%, however, there are other reports providing much higher rates. The majority of the conducted studies investigating ICDs focus mainly on pharmacological factors, however, from a psychological perspective, there is yet enough room for investigation. In order to address the above issues, we designed a two-part study. *Materials and Methods:* First, we aimed to identify the incidence of ICD and related behaviors in a cohort of 892 Greek PD patients. Second, we administered a comprehensive battery of psychometric tools to assess psychological factors such as personality dimensions, quality of life, defenses, coherence, and resilience as well as to screen general cognitive capacity in PD patients with ICD manifestations. *Results:* With regard to the first part, we identified ICD manifestations in 12.4% of the patients. Preliminary findings from the second part indicate elevated activity, rather than impulsivity, as well as interrelations between several variables, including measures of activity, coping mechanisms, and quality of life. *Conclusions:* We present a working hypothesis for the contribution of high activity channeled to specific behavioral patterns through specific coping mechanisms, concerning the emergence of ICDs and related behaviors in PD, and further stress the importance of compulsivity rather than impulsivity in this process.

## 1. Introduction

Problematic behaviors with impulsive compulsive addictive characteristics such as pathological gambling, binge eating, compulsive buying, and hypersexuality, most commonly described as Impulse Control Disorders (ICDs), as well as other problematic behavioral patterns like the self-regulated addiction-like overconsumption of dopaminergic treatment described as Dopamine Dysregulation Syndrome (DDS) and other impulsive-compulsive behaviors (ICBs) like punding and hobbyism, have been shown to be common non-motor complications of Parkinson’s Disease (PD) [1]. ICDs have been shown to have a great impact on PD patients, with severe psychosocial consequences, resulting in poor quality of life [2,3] and increased caregiver burden [4].

During the last two decades, several studies have investigated the prevalence of ICDs and ICBs among PD patients. Large-scale studies report a prevalence of more than 10% in American and Asian populations [1,5], while there are national studies (i.e., the sample is collected from a single country) indicating higher prevalence rates, exceeding 25% [6,7,8]. In general, ICD/ICB frequency estimates range from approximately 10 to 60% in PD, see also [1,9,10].

Based on the vast majority of published studies, the emerging consensus points to male sex, young age, early onset, existence of anxiety and depression, impulsivity, and novelty-seeking personality traits as the main non-pharmacological factors associated with ICDs [11,12]. There are also indications that the establishment of those behaviors may precede the onset of the disease, and that they may be associated with familial/personal history of substance use disorders or gambling. The main ICD-associated pharmacological factor is the intake of dopamine agonists (DA), and both levodopa augmentation and apomorphine seem to be correlated with DDS [13,14]. It has been argued that DA intake, in combination with genetic risk, is a prerequisite for the emergence of ICDs [15]. There are, however, studies which have shown distinct ICDs/ICBs in drug-naïve patients, e.g., [16], thus underlining the necessity of assessment in early stages.

Most researchers are investigating pharmacological, clinical, and demographic factors and their relationship to ICDs and ICBs in PD (for a review see [12,17]). There are also studies investigating possible links between ICDs/ICBs and personality and specific impulsivity-related traits. Consequently, the need for a more thorough assessment of traits in PD patients exhibiting ICD symptomatology or merely isolated ICBs, becomes evident.

With regard to etiology, there is a significant number of studies focusing on the understanding of the underlying mechanisms of the aforementioned behaviors for a review, see [18]. Most of the explanatory models commonly involve the reward–punishment circuits, especially dysregulation in the mesocorticolimbic dopamine pathways, e.g., [19]; for a recent research study providing evidence about the involvement of specific white matter tracts in reward and punishment processing see [20] and D3 receptors. Other DA-related genetic variants, such as DRD1 and DRD2, as well as genes associated with other neurotransmitter systems, such as *OPRK1* and *HTR2A*, have been implicated in ICDs [21,22,23], but see also [24]. In contrast, studies focusing on psychological factors are relatively scarce and mostly limited to the assessment of specific personality dimensions such as those falling under the umbrella of the impulsivity construct [25,26].

Overall, while there is much research on ICDs and ICBs in PD, the exact role of psychological factors possibly contributing to such behavioral patterns is yet to be fully understood. This study presents the first findings from an ongoing project carried out at Eginition Hospital (i.e., “Investigation of non-motor symptoms in Parkinson’s Disease”). In order to do that, we followed a two-step process. We initially report descriptive data about the frequency of ICDs and ICBs in a large cohort of Greek PD patients. Then, we present preliminary results derived from a subsample of the cohort, following a psychologically oriented approach to investigate possible factors associated with such non-motor symptomatology. The rationale of our ongoing project is to highlight the importance of psychological factors which could serve as complementary indices in the framework of a future explanatory model that would eventually provide adequate hypotheses for the multifaceted phenomenon of ICDs and ICBs in PD.

## 2. Methodology

### 2.1. Archival Sample

A total of 892 PD cases were examined through an archival study of the records of the Specialized Movement Disorders Outpatient Clinic of the First Neurology Department at Eginition Hospital, National and Kapodistrian University of Athens. Diagnosis of PD was established by senior neurologists specialized in movement disorders on the basis of formal diagnostic criteria [27,28], after a comprehensive neurological, cognitive, and neuroimaging evaluation, including Unified Parkinson’s Disease Rating Scale (UPDRS) for motor/non-motor assessment and MoCA (Montreal Cognitive Assessment) Scale. ICDs and/or ICBs were identified on the basis of a detailed clinical interview. During this clinical interview, the clinician asked questions investigating dopamine dysregulation syndrome, nutrition, sexual activity, gambling, money spending, leisure time activities, and hobbies. Of particular interest was the identification of behavioral patterns indicating ICDs/ICBs. Impulse control disorders and behavioral addictions are mainly characterized by repetitive lack of control, i.e., inability to resist despite the resulting severe psychosocial and/or financial consequences, as well as reduction of functionality in certain domains of daily living, including, among others, working, studying, and personal relations [29]. In this diagnostic framework, the clinician meticulously gathered information by thoroughly interviewing the patient in order to record specific behavioral patterns of interest as ICB or ICD manifestations. When the patient was escorted by a caregiver, the latter was also interviewed about the course of the disease, potential difficulties, and changes in everyday life’s activities, as well as for observed ICD/ICB signs. The above information was included in the patients’ files that were retrospectively and individually examined by our group. The outcome of this case-by-case investigation resulted in the provided frequencies of ICDs/ICBs presented in this study.

### 2.2. Preliminary Study’s Sample

A total of 62 PD patients have been contacted and, so far, 30 have agreed to participate in the study. All participants were sampled from the project “Investigation of non-motor symptoms in Parkinson’s Disease” conducted at Eginition Hospital in Athens, School of Medicine, Greece (research protocol approval 7Υ6Ψ46Ψ8Ν2-AOΒ, 2017).

### 2.3. Psychometric Tools

Patients were assessed individually by a licensed psychologist at the 1st Neurology Department within a single session, with a comprehensive battery of psychometric tools described below.

Two neuropsychological tools were used. To assess general cognitive capacity, we administered the Greek version of the MoCA, which is a screening test for visuospatial abilities, memory, attention and concentration, executive functions, and language and orientation [30,31]. The Greek version of this tool has been shown to exhibit excellent psychometric properties, with specificity and sensitivity ranging from 92.3 to 100 and 97.6 to 100, respectively (for the diagnosis of dementia) [31]. This screening test has already been used in research with PD patients, e.g., [32]. In order to obtain an index of executive function, the Controlled Oral Word Fluency test (COWF) was administered. COWF includes two parts: a semantic subscale, in which the participant is asked to generate words falling under a specific semantic category, and a phonemic subscale, in which the participant is asked to generate words starting with a specific initial letter; for both subscales, word generation is measured within the time frame of 60 s [33]. This is the Greek version of the Controlled Oral Word Association Task, with an interrater reliability score of 0.91 [33], and has been widely used in various Greek-speaking clinical populations.

Patients’ personality dimensions were evaluated with the Greek version of the Zuckerman–Kuhlman Personality Questionnaire (ZKPQ) [34]. This tool exhibits very good psychometric properties, with test–retest reliability ranging from 0.79 to 0.89 (depending on the subscale examined), indices of internal consistency (Cronbach’s alpha) ranging from 0.64 to 0.87 (depending on the subscale examined), and adequate validity [34]. The ZKPQ measures 5 dimensions: Activity, Aggression-Hostility, Neuroticism-Anxiety, Sociability, and Impulsive Sensation Seeking [35]. This tool has been previously used to assess personality traits of PD patients concerning impulse control disorders [36]. To assess signs of psychopathology, we used the Greek version of the Symptom Checklist-90 (SCL-90) [37], which includes 9 major categories of symptoms, namely Somatization, Obsessive Compulsive, Interpersonal Sensitivity, Depression, Anxiety, Hostility, Phobic Anxiety, Paranoid Ideation, and Psychoticism, as well as 7 additional items related to sleep, appetite, guilt, and thoughts of death [38,39]. There are several authors reporting that this tool is reliable and that it shows adequate indices of convergent and criterion-related validity, e.g., [37,40]. Moreover, the Greek version of the Defense Style Questionnaire–88 (DSQ-88) [41] was administered to evaluate ego defense mechanisms comprising 4 defense styles, namely, “maladaptive action” style, “image-distorting” style, “self-sacrificing”, and “adaptive” style (Bond et al., 1983). This tool has been shown to exhibit adequate test–retest reliability, ranging from 0.77 to 0.88, and internal consistency indices ranging from 0.66 to 0.82 (depending on the subscale examined) [41].

Additionally, we administered the Connor–Davidson Resilience Scale (CD-Risk) [42,43] to measure the ability to bounce back in the face of adversity, a tool exhibiting excellent internal consistency and test–retest reliability, as well as adequate convergent validity [43]. Moreover, the Sense of Coherence Scale (SOC) [44,45] was used to assess meaningfulness, manageability, and comprehensibility of stressful events. Studies focused on the psychometric properties of the scale report very good test–retest reliability and excellent internal consistency [45,46]. Finally, the World Health Organization/Quality of Life-Bref (WHOQOL-BREF) [47] was administered to assess quality of life. Reported internal consistency was found to be within 0.65–0.87, convergent validity was overall satisfactory, and test–retest reliability was judged to be excellent [47]. This study has been approved by the Ethics Committee of Eginition Hospital (research protocol approval ID: 7Υ6Ψ46Ψ8Ν2-AOΒ).

## 3. Results

### 3.1. Frequency of ICDs/ICBs Based on the Archival Study

From the total cohort of 892 patients, we identified 111 individuals (12.4%) demonstrating ICDs and/or ICBs. Table 1 shows the demographic and clinical information for these patients. Males exhibited gambling and hypersexuality more frequently compared to females (x^2^ = 9.98, *p* < 0.01 and x^2^ = 8.72, *p* < 0.01, for gambling and hypersexuality, respectively), while the opposite pattern was observed with regard to compulsive binge eating (x^2^ = 14.50, *p* < 0.001). Box 1 shows examples of behavioral patterns compatible with ICD/ICB.

Box 1 **Examples selected from clinical interviews illustrating ICD/ICB-related behavioral patterns.**

***Binge eating***

One patient mentioned that she seems to be always hungry and never full. Another patient used the following phrases to describe her eating habits “I binge eat. I eat a lot of snacks […] I spend a lot of time every day thinking about food […] I can’t control myself when it comes to eating […] I lie when it comes to eating.” Another patient’s wife said to the clinician that her husband “devours everything, all day long, every sweet that he can find.”

***Gambling***

One patient mentioned that he gambles for 12 consecutive hours on the computer and stops after having lost everything. As the patient himself remarks, “I don’t want to do anything else. When I am on the computer, I lose track of everything else around me and time just passes by.” Another patient’s wife showed up repeatedly at the clinic desperate for help as her husband had went missing and had also removed her name from their joined bank account. When his daughter came with him to his scheduled appointment, she claimed that her father had gambled a lot of money at the casino.

***Compulsive buying/Hoarding***

There was one patient who used to collect bottle caps. For many years, he would collect thousands of caps at his home. He said, “I see bottle caps on the streets and it bothers me, I think that there will be no caps left.” Another patient admitted to hoarding large quantities of snacks. A third patient said that she was constantly buying ladles.

#### 3.1.1. Frequency of ICDs/ICBs and Patients Identified with a Single ICD/ICB

**Figure 1:**
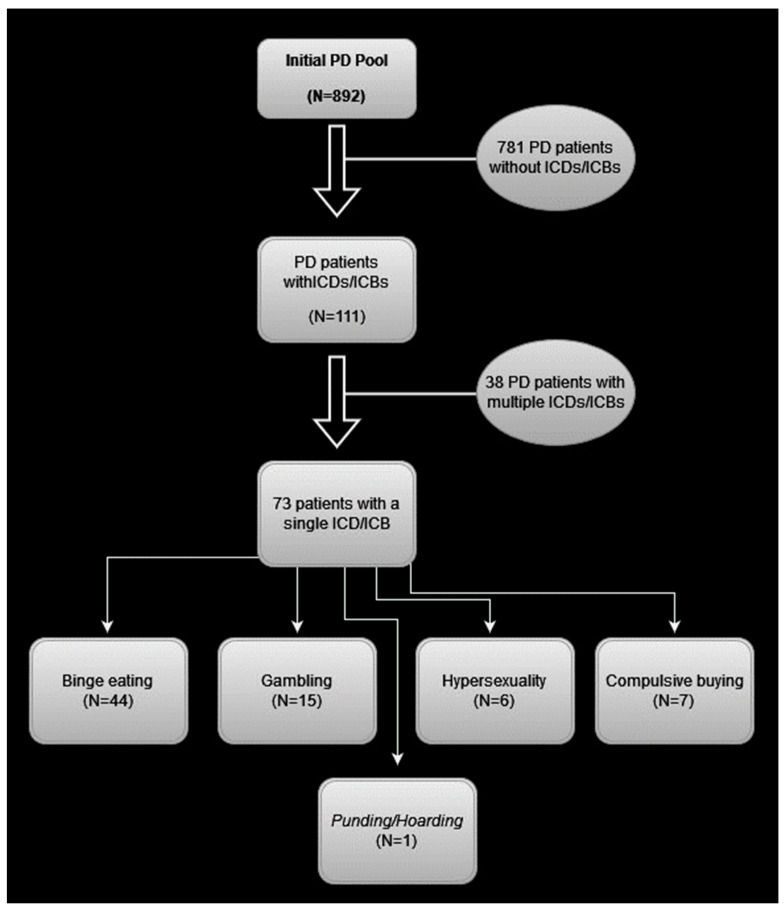
Flowchart showing ICD/ICB frequency for the patients exhibiting a single ICD/ICB.

Seventy-two patients manifested a single ICD, while 38 patients manifested more than one and one patient exhibited a single ICB, i.e., punding/hobbyism. The most frequent ICD was binge eating, demonstrated by 80 patients (72.1% of the ICD/ICB subgroup; 9% of the total cohort), followed by compulsive buying (35 patients; 31.5% of the ICD/ICB subgroup; 3.9% of the total cohort), then gambling (26 patients; 23.4% of the ICD/ICB subgroup; 2.9% of the total cohort), and lastly, hypersexuality (17 patients; 15.3% of the ICD/ICB subgroup; 1.9% of the total cohort). Of the 72 patients who manifested a single ICD, 44 exhibited binge eating, 15 patients exhibited gambling, 7 patients exhibited compulsive buying, and 6 patients exhibited hypersexuality (see Figure 1).

#### 3.1.2. Patients Identified with Multiple ICDs/ICBs

**Figure 2:**
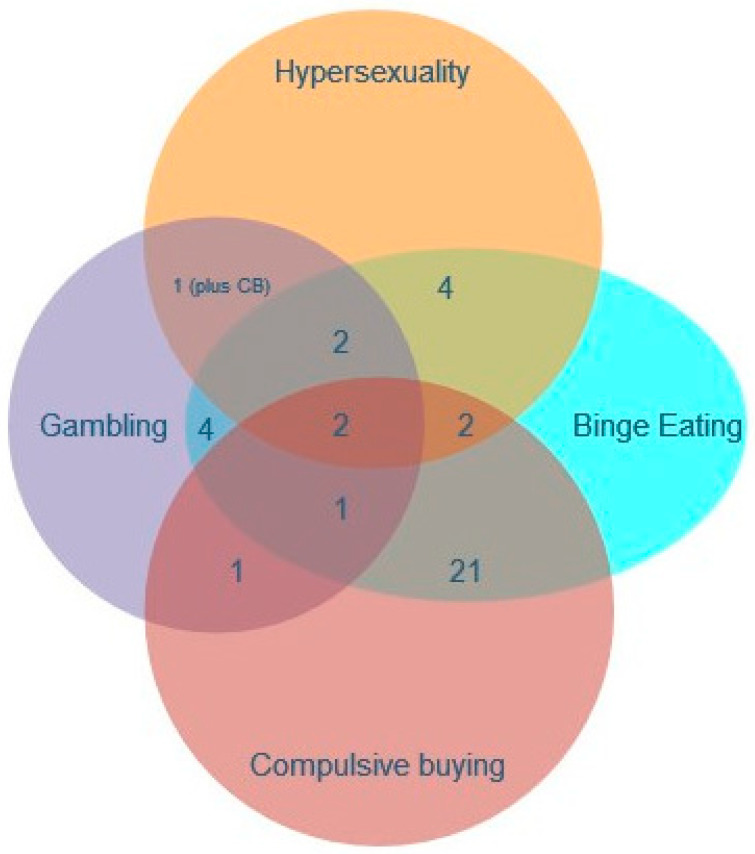
Illustration of the frequency of multiple co-existing ICDs. Only patients with more than one ICD are included. Numbers correspond to the N of patients.

With regard to the remaining 38 patients, the most frequent overlap was observed between binge eating and compulsive buying (18.9% of the ICD/ICB subgroup; 2.3% of the total cohort), followed by two equal subgroups of 4 patients demonstrating binge eating + gambling and binge eating + hypersexuality (3.6% of the ICD/ICB subgroup; 0.4% of the total cohort each), then three equal groups of two patients exhibiting gambling + hypersexuality + binge eating, gambling + hypersexuality + binge eating + compulsive buying, and hypersexuality + binge eating + compulsive buying (1.8% of the ICD/ICB group and 0.2% of the total cohort each), and lastly, there was one patient with gambling + hypersexuality + compulsive buying, one with gambling + binge eating + compulsive buying, and one with gambling + compulsive buying (0.9% of the ICD/ICB subgroup; 0,1% of the total cohort for each case) (for details, see Figure 2).

#### 3.1.3. Specifics on the ICBs and Non-Motor Symptoms in General

Of the 111 patients, 22 individuals (19.8% of the ICD/ICB subgroup; 2.5% of the total cohort) also manifested other ICBs, while, as mentioned above, there was one patient who exhibited only punding/hobbyism. The most prominent ICBs were hobbyism (9 patients) and hoarding (5 patients), while 11 patients were identified as demonstrating DDS. Mean disease duration upon initial manifestation of ICD/ICB symptomatology was 6.78 years (SD: 4.83 years). Pharmacological archival data were available for 97 cases, which were treated with DA at the time of ICD/ICB manifestation. With regard to other non-motor symptomatology in the ICD/ICB subgroup, 65 patients suffered from sleep-related disorders, such as REM Sleep Behavior Disorder and other parasomnias. Moreover, concerning psychiatric manifestations, 39 patients exhibited psychotic symptoms with the most prominent being visual hallucinations (24 patients), and 29 patients were diagnosed with depression and were under anti-depressant medication.

### 3.2. Preliminary Results from the 30-Patient Sample

This preliminary sample consisted of 16 males and 14 females, with a mean age of 68 years (SD: 7.99 years), mean age of disease onset at 55.5 years (SD: 9.93 years), and mean age of manifestation of ICDs and/or ICBs at 62.71 years (SD: 6.02 years).

#### 3.2.1. Impulsivity vs. Activity

**Figure 3:**
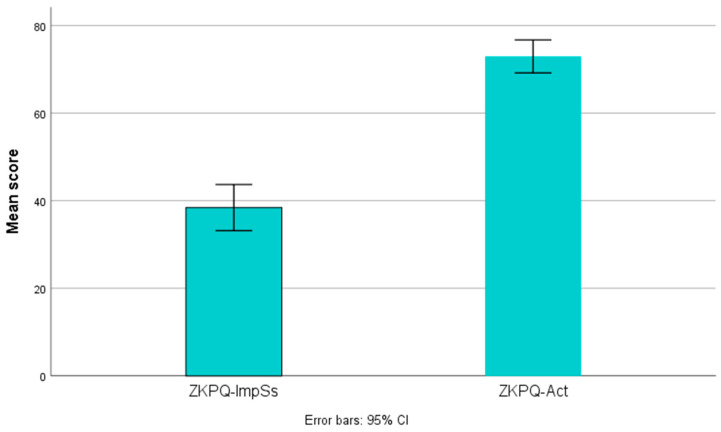
Error bar chart showing mean scores on the ZKPQ subscales for activity and impulsivity/sensation seeking.

**Figure 4:**
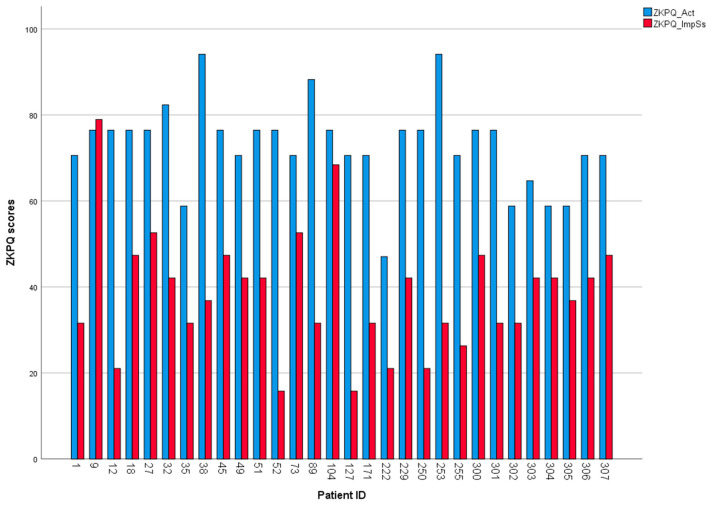
Bar chart showing individual data on ZKPQ values for activity and impulsivity/sensation seeking.

Table 2 presents the scores on psychometric tools. After initial conduction of descriptive statistics, we observed the discrepancy between mean values of the impulsiveness-sensation seeking trait (which was predetermined as a variable of interest for the scope of our study) and the activity trait. Therefore, we decided to compare these two variables and carry out a case-by-case investigation. Paired-samples *t*-test revealed that the observed difference is statistically significant [t(29) = 11.695, *p* < 0.001)] (see Figure 3). Moreover, the case-by-case investigation revealed that almost all patients exhibited this discrepancy individually (see Figure 4).

#### 3.2.2. Associations between Psychological Variables

We conducted correlation analyses to assess possible relationships between activity and several targeted variables of interest related to quality of life and defense mechanisms. Notably, activity was correlated with subjective overall quality of life (rho = −0.615, *p* < 0.01), while marginal associations were found with DSQ sublimation (rho = 0.486, *p* = 0.056) and task orientation (rho = 0.439, *p* = 0.089). Significant correlations were also found between CD-RISK and WHO psychological dimension (rho = 0.570, *p* < 0.05), as well as sense of coherence and subjective overall health (rho = 0.539, *p* < 0.05). Moreover, CD-RISK was significantly correlated with sublimation (rho = 0.556, *p* < 0.05) and marginally with task orientation (rho = 0.483, *p* = 008). It should be noted that we characterize some of the above correlations as “marginal” not only based on their *p*-values’ proximity to the predetermined level of significance (α = 0.05) but also on the basis of the correlation coefficients which could be interpreted as being in the moderate range. In any case, these correlations are not statistically significant, and we consider them at best as indicative of a possible trend. The trend will be confirmed or rejected with additional data of the present ongoing study in future reports. Finally, there was no significant correlation between the activity variable and L-DOPA equivalent daily dose (rho = 0.126, *p* = 0.653).

## 4. Discussion

### 4.1. On the Frequency of ICDs/ICBs in PD

Our findings showed that 12.4% of our PD cohort exhibited ICDs/ICBs, with the greatest proportion of that group demonstrating a single ICD. The available data reported in the relevant literature are not particularly conclusive concerning the prevalence of such behaviors in PD. For example, in their recent review, Zhang and colleagues [13] reported a rather wide range of 3.5–43%. Other reviews reported a much narrower range; for example, Wu, Politis, and Piccini [48] argued that the prevalence is between 5.9% and 13.9% and Ambermoon and colleagues [49] reported a range between 3.5 and 13.6%. In sum, such reviews have clearly illustrated the discrepancy between studies attempting to describe the prevalence of ICDs in PD. With regard to ICBs, Rhode and colleagues [10] reported a frequency of 60% in a sample of 739 German patients. Our findings generally fall within the reported range and are similar to data published by other scholars, such as Weintraub et al. [1] who showed a prevalence of 13.6% in their large-scale study, and Smith, Xie, and Weintraub [50] who reported a prevalence of 8% which increased up to 25% in their longitudinal study. The above-mentioned variability across studies has been clearly illustrated by Molde and colleagues [9] in their recent meta-analysis, which showed a rather wide range of 6.12% to 58.3% concerning the frequency of ICDs in PD samples. The observed discrepancies could be attributed to several factors which would differentially affect the cohorts of such studies, including psychiatric comorbidities, clinical variables, and pharmacological treatments. As stated in the Introduction, there are several pharmacological and non-pharmacological factors associated with the development of ICDs in PD [11,12]. Since several of such factors (e.g., age or gender of participants, disease duration, pharmacological augmentation) greatly vary between research reports, the corresponding samples, and subsequently the studies’ findings may not be directly comparable; see also [11] for the controlling for such heterogeneity across studies in a meta-analysis. Another source of variability could be sample size, which may affect the statistical power of the study when conducting formal analyses or, especially in cases of small samples, may lead to bias when estimating frequencies of ICDs. Finally, even though most studies followed standard clinical assessment guidelines for PD diagnosis, they used different means of ICD/ICB identification, and therefore, frequency estimations may vary across reports.

The most common addictive behavior was compulsive binge eating, followed by compulsive buying, then gambling, and lastly, hypersexuality. In general, our findings are in accordance with other studies for a review, see [14], even though the relative frequencies observed in our sample differed from other reported data [1]; for a review, see [51]. It should be noted that in the general population, the prevalence of compulsive buying is 4.9% [52]; see also [53], while older studies have provided estimates of up to 16% [54]. The prevalence of binge eating ranges from 0.7% to 1% [55] and lifetime prevalence of pathological gambling is between 0.4 and 1% [56]; see also [57]. As for hypersexuality, even though there is no classification for a corresponding disorder in DSM5, and therefore, epidemiological studies are generally lacking, a safe prevalence rate is estimated at the range of 3–6% [58]. The above prevalence rates seem to depend on sociodemographic factors, e.g., [55,56]. This is also reflected in our results, since males exhibited gambling and hypersexuality more frequently compared to females, while the opposite pattern was observed with regard to compulsive binge eating.

### 4.2. Psychological Factors Associated with ICDs/ICBs in PD

According to the preliminary results derived from the 30-patient sample, the lowest score on the WHO-BREF corresponded to the individuals’ subjective view of their overall health. Regarding the basic dimensions of the questionnaire, it was not surprising that the lowest score was related to physical health, in contrast to the mean score associated with the environmental domain. Overall, the WHO-BREF results indicate that PD may have a differential detrimental effect on various aspects of health and quality of life, and further highlight the necessity of including such psychometric measures in the assessment of patients suffering from this disease, which after all seems to result in a rather complex constellation of intercorrelated difficulties in physical, psychological, social, and other aspects of everyday life see also [59,60]. It should also be noted that even though only a small portion of individuals has been assessed so far with the extensive battery described in the Methodology, the results from the SCL-90 indicated signs of binge eating and sleep-related non-motor symptoms among our participants.

SOC scores were widely distributed, and given the small sample of the preliminary study, no robust conclusions can be drawn at this point. However, it should be noted that there were individual patients whose score indicated an impaired sense of coherence on the basis of previously reported normative data from healthy controls [44,46], an observation which may be related to the reported deleterious effect of a low sense of coherence on coping strategies. Pusswald and colleagues [61] compared PD patients with people who had other chronic non-neurological diseases and found that the mean SOC score of the PD group was significantly lower. The authors argued that lower SOC scores could be associated with less effective coping strategies. Therefore, we hypothesize that those individuals with SOC scores below a predetermined cutoff might face relevant difficulties in adaptation. This issue will be clarified in a subsequent study with a greater group; however, we would like to draw the attention of the clinicians to this matter and stress the importance of taking into consideration such measures when treating a patient.

The most interesting finding was that of a high level of activity, as revealed by the case-by-case investigation. This is further supported by contrasting the mean activity score of our PD group with mean scores on the same subscale of ZKPQ reported in other studies, involving healthy but also pathological Greek populations [34,62], as well as PD patients without ICDs [36].

Few studies have tried to illustrate the PD personality. The most common characteristics seem to be: introversion, inflexibility, high neuroticism, low novelty seeking, and high harm avoidance, where the aforementioned have similarities to obsessive-compulsive personality disorder [63,64,65]. In an older review, Todes and Lees [66] present several early studies which described PD premorbid personality. Even though the authors’ explanatory rationale is within a framework of theoretical psychiatry, they share common intellectual ground with the above-mentioned recent papers. In this sense, they argued that specific premorbid personality types may have a causal relationship with the development of PD, with PD patients being premorbidly inflexible, over-controlling, with a predisposition for depression and suppressed aggression and sexual drive. Our findings could be interpreted in the context of the above theoretical framework. In our sample, impulsivity and sensation seeking were not found to be elevated, in contrast to activity which, for the vast majority of patients, was at a much higher level. The personality dimension of activity contains two facets, reflecting the individual’s need for general and work activity, both linked to persistence, restlessness, perfectionism, and perseverance [34]. In order to explain our participants’ strong need for activity, we examined possible correlated factors. We investigated possible correlations between activity scores and dopaminergic medication (L-Dopa equivalent daily dose), however, the analyses yielded null results. This finding seems to be in line with recent studies which show ICDs/ICBs in drug-naïve patients. For example, Smith and colleagues [50] report the presence of addictive behaviors in 84 individuals out of a cohort of 418 PD patients before the initiation of pharmacological augmentation; see also [16]. Even though other factors, such as possible effects of antidepressant medication, could not be examined in the present study, our findings overall indicate that the elevated level of the specific dimension reflects a need for general and work activity as well as persistence.

Although the finding of consistently high activity relative to impulsivity has not been particularly discussed in the literature, the idea that the latter trait may not play a central role in such behaviors exhibited by specific clinical populations, including PD patients, is nihil novi. Despite the traditional view that ICDs may mainly stem from impulsivity, there are several studies which question its relation to those behaviors, as this particular personality dimension includes miscellaneous components and often overlaps with compulsivity [67,68,69,70]. In accordance with this notion, we argue that in our study, the compulsive rather than the impulsive element seems to be the most crucial factor.

### 4.3. A Working Hypothesis on Addictive Behaviors of PD Patients

Since this is an ongoing study and the results derived from psychometric evaluation are preliminary, we are reluctant to draw any final conclusions, and therefore, we will present a working hypothesis on addictive behaviors demonstrated by PD patients. The development of such addictive behaviors as an attempt to overcome anxiety and depression has already been proposed in the past. There is also comorbidity of addictive behaviors with mood and anxiety disorders, which seems to be of a bidirectional nature. For example, some individuals approach pathological gambling in a failed attempt to overcome negative feelings or stressful events [71]. Moreover, binge eating is strongly linked to anxiety, depression, and stress, in the sense that people suffering from binge eating may demonstrate food overconsumption as a compulsive maladaptive way to escape and seek relief from anxiety [72,73,74]. The same has been argued for compulsive buying as well; for a review, see [75].

Our patients scored higher on the Adaptive Defenses profile of DSQ, which is an indication of an individual seeking a healthy and mature way to overcome anxiety. In particular, high scores on defense subscales such as sublimation and task orientation indicate that anxiety resolution may be achieved or at least attempted by developing adaptive coping mechanisms. While acknowledging that the items examining those mechanisms in DSQ are relatively few, our participants’ scores are high enough to be accepted as a clear indication for the implementation of those defenses. From a strictly psychological perspective, our results support the notion that the manifested behaviors may be recruited by the Parkinsonic subject as an attempt to overcome the psychological burden of the disease. Furthermore, from an economic perspective, the redundant amount of activity may give rise to the compulsive drive that maintains those behaviors. As the motor symptoms progress over time, the patient attempts to regress in a premorbid situation, focuses on basic life aspects, which can be considered rewards, like nourishment and sexual activity, hoarding of industrial products, money that could be spent or gathered through gambling, and the creation of artifacts that will remain through hobbyism, while they are aware of the fact that their abilities will decrease over the course of the disease and that their motor capacity is ephemeral. Although these behaviors are not per se pathological, they are compulsively elevated to a pathological degree resulting in a de novo disorder other than PD. Correlated factors such as young age, early onset, and the presence of depression may also hint towards that hypothesis.

## 5. Strengths, Limitations, and Future Directions

Even though the archival part of the study may be considered large-scale, the results from the second part of our project are derived from a relatively limited number of patients and therefore should be considered preliminary. In this context, another limitation is the lack of control groups, i.e., a group of PD patients without the manifestation of the examined behaviors and a matched group of healthy controls. Furthermore, our participants were not classified under UPDRS or Hoehn and Yahr Scale stages. Another limitation of the present study is that we did not manage to quantify addictive behaviors. However, our study offers insight into the prevalence of ICDs and ICBs in the Greek PD population. In addition, an element of novelty is that a comprehensive battery of psychometric tools examining psychological factors such as defenses, coherence, and resilience that have been previously used in populations with other chronic diseases, were utilized in PD patients with certain behavioral patterns. Most importantly, our findings highlight the possible role of a personality trait other than impulsivity, namely, activity, in ICDs/ICBs in PD, and further provide indications in compliance with older theoretical perspectives on compulsive behaviors as a failed attempt to overcome the burden of the disease. Future studies should examine the above hypothesis in larger populations, including drug-naïve patients of different age, onset, and chronicity of PD. The multifaceted phenomenology of ICDs/ICBs in PD requires a multidisciplinary approach, which should unequivocally be accomplished by multivariate statistical processes applied in large and diverse cohorts in order for the demonstration of addictive behaviors of such patients to be elucidated and eventually for their etiology to be fully understood.

## Figures and Tables

**Table 1:** Demographic and clinical information for the 111 patients of the archival study.

Sex	69M/42F
Marital status	Married: 86; Single: 12; Divorced: 4; Widowed: 9
Parenthood	81 individuals with children
Age of onset—Mean (SD)	56.23 (8.70)
Age at manifestation of ICDs—Mean (SD)	62.29 (7.17)
Psychiatric manifestations	29 patients diagnosed with depression 39 patients with psychotic manifestations
Sleep disturbances	65 patients with RBD/parasomnias
Family neurological history	*n* = 26
Comorbidities with systemic diseases	Thyroid (*n* = 26); Heart disease (*n* = 19); Diabetes (*n* = 8)

**Table 2:** Scores on the psychometric tools for the 30-patient sample.

Psychometric Tool	Min	Max	Mean	SD
ZKPQ-ImpSs	15.79	78.95	38.42	14.11
ZKPQ-NAnx	5.26	89.47	48.07	27.32
ZKPQ-AggHost	5.88	70.59	30.59	18.76
ZKPQ-Act	47.06	94.12	72.94	10.08
ZKPQ-Sy	5.88	76.47	44.12	16.76
DSQ-As	2.61	7.84	5.79	1.41
DSQ-MAs	2.33	5.45	3.64	0.90
DSQ-SSs	2.61	6.31	4.38	1.05
DSQ-IDs	1.38	5.41	3.57	1.13
SCL-GSI	0.22	1.88	0.89	0.48
CD-RISK	53.00	96.00	76.25	13.39
SOC	104.00	177.00	143.13	20.30
MoCA	19.00	29.00	24.71	2.54
COWF-S	25.00	63.00	44.59	10.56
COWF-Ph	16.00	42.00	29.82	8.45
WHO-QOL—Physical	25.00	85.71	56.5124	18.09
WHO-QOL—Psychological	41.67	95.83	64.22	15.80
WHO-QOL—Social	33.00	83.33	57.8229	14.31
WHO-QOL—Environmental	50.00	90.63	70.96	11.89
WHO-QOL—SubjQOL	25.00	100.00	60.29	19.88
WHO-QOL—SubjH	25.00	100.00	51.47	25.72

ZKPQ-ImpSs: Zuckerman–Kuhlman Personality Questionnaire—Impulsiveness/Sensation Seeking trait; ZKPQ-NAnx: Zuckerman–Kuhlman Personality Questionnaire—Neuroticism/Anxiety trait; ZKPQ-AggHost: Zuckerman–Kuhlman Personality Questionnaire—Aggressiveness/Hostility trait; ZKPQ-Act: Zuckerman–Kuhlman Personality Questionnaire—Activity trait; ZKPQ-Sy: Zuckerman–Kuhlman Personality Questionnaire—Sociability trait; DSQ-As: Defense Style Questionnaire–88—Adaptive style; DSQ-MAs: Defense Style Questionnaire–88—Maladaptive/Action style; DSQ-SSs: Defense Style Questionnaire–88—Self-sacrificing style; DSQ-IDs: Defense Style Questionnaire–88—Image Distortion style; SCL-GSI: Symptom Checklist-90—General Severity Index; CD-RISK: Connor–Davidson Resilience Scale; SOC: Sense of Coherence Scale; MoCA: Montreal Cognitive Assessment; COWF-S: Controlled Oral Word Fluency—Semantic subscale; COWF-Ph: Controlled Oral Word Fluency—Phonemic subscale; WHO-QOL—Physical: World Health Organization/Quality of Life-Bref—Physical Factor; WHO-QOL—Psychological: World Health Organization/Quality of Life-Bref—Psychological Factor; WHO-QOL—Social: World Health Organization/Quality of Life-Bref—Social Factor; WHO QOL—Environmental: World Health Organization/Quality of Life-Bref—Environmental Factor; WHO-QOL—SubjQOL: World Health Organization/Quality of Life-Bref—Overall Subjective Quality of Life Factor; WHO QOL—SubjH: World Health Organization/Quality of Life-Bref—Overall Subjective Health Factor.

## Data Availability

Data are available upon reasonable request to the corresponding author.
